# *DREF* Genetically Counteracts *Mi-2* and *Caf1* to Regulate Adult Stem Cell Maintenance

**DOI:** 10.1371/journal.pgen.1008187

**Published:** 2019-06-21

**Authors:** Benjamin Angulo, Shrividhya Srinivasan, Benjamin J. Bolival, Gonzalo H. Olivares, Allyson C. Spence, Margaret T. Fuller

**Affiliations:** 1 Department of Biology, Stanford University, Stanford, CA, United States of America; 2 Department of Developmental Biology, Stanford University School of Medicine, Stanford, CA, United States of America; 3 Oncology Biomarker Development, Genentech Inc, South San Francisco, CA, United States of America; 4 Department of Neuroscience, Faculty of Medicine, Universidad de Chile, Santiago, Chile; 5 Women’s Cancer Center, Stanford University Hospital, Stanford, CA, United States of America; 6 Department of Genetics, Stanford University School of Medicine, Stanford, CA, United States of America; Carnegie Institute of Washington, UNITED STATES

## Abstract

Active adult stem cells maintain a bipotential state with progeny able to either self-renew or initiate differentiation depending on extrinsic signals from the surrounding microenvironment. However, the intrinsic gene regulatory networks and chromatin states that allow adult stem cells to make these cell fate choices are not entirely understood. Here we show that the transcription factor *DNA Replication-related Element Factor* (*DREF*) regulates adult stem cell maintenance in the Drosophila male germline. A temperature-sensitive allele of *DREF* described in this study genetically separated a role for *DREF* in germline stem cell self-renewal from the general roles of *DREF* in cell proliferation. The *DREF* temperature-sensitive allele caused defects in germline stem cell self-renewal but allowed viability and division of germline stem cells as well as cell viability, growth and division of somatic cyst stem cells in the testes and cells in the Drosophila eye. Germline stem cells mutant for the temperature sensitive *DREF* allele exhibited lower activation of a TGF-beta reporter, and their progeny turned on expression of the differentiation factor Bam prematurely. Results of genetic interaction analyses revealed that *Mi-2* and *Caf1/p55*, components of the Nucleosome Remodeling and Deacetylase (NuRD) complex, genetically antagonize the role of *DREF* in germline stem cell maintenance. Taken together, these data suggest that *DREF* contributes to intrinsic components of the germline stem cell regulatory network that maintains competence to self-renew.

## Author summary

Many adult tissues are maintained throughout life by the dual ability of adult stem cells to produce progeny that either self-renew or differentiate to replace specialized cells lost to turnover or damage. Although signals from the surrounding microenvironment have been shown to regulate the choice between self-renewal and onset of differentiation, the intrinsic gene regulatory programs that set up and maintain this bipotential state are not well understood. In this report we describe antagonistic components of an intrinsic stem cell program important for maintaining the balance between self-renewal and differentiation in Drosophila male germline adult stem cell lineage. We identified a temperature-sensitive mutant in the transcription factor *DNA Replication-related Element Factor* (*DREF*) gene that disrupts the ability of germline stem cells to self-renew, but not stem cell viability, ability to divide or differentiate under the same conditions. *DREF* mutant germline stem cells showed defects in the TGF-beta signaling pathway, a pathway that is critical for maintaining the stem cell population. Genetic interaction analyses revealed that *Mi-2* and *Caf1/p55*, components of the Nucleosome Remodeling and Deacetylase complex genetically antagonize the role of *DREF* in germline stem cell maintenance. We propose that DREF contributes to a transcriptional environment necessary for maintaining a bi-potential stem cell state able to properly respond to extrinsic niche signals.

## Introduction

Adult stem cells maintain tissues during the lifetime of an organism by replenishing short-lived differentiated cells such as in the skin, intestinal epithelium and blood. Adult stem cells also give rise to differentiated cells upon injury in tissues such as skeletal muscle and lung. To maintain tissue homeostasis, daughter cells produced by adult stem cell divisions must make the critical cell fate decision between self-renewal and the onset of differentiation. Deviation from the tightly regulated balance between these alternate fates may result in poor tissue maintenance or cancerous growth of poorly differentiated precursor cells[1]. Adult stem cells are thus in a bipotential state, able to self-renew or to initiate differentiation in response to extrinsic signals from the surrounding microenvironment[2,3]. This bipotential state relies on intrinsic transcriptional and chromatin programs that dictate how stem cells respond to external signals from the niche.

Here we show that in Drosophila male germline adult stem cells, the transcription factor DNA Replication-Related Element Factor (DREF) and members of the Nucleosome Remodeling and Deacetylase (NuRD) complex, Mi-2 and Chromatin Assembly Factor 1 (Caf1, also known as p55), act antagonistically to regulate the balance between germline stem cell self-renewal and differentiation. In the adult testis, two populations of stem cells, germline stem cells (GSCs) and somatic cyst stem cells (CySCs), reside adjacent to a group of post-mitotic somatic cells called the hub. The hub cells and the CySCs provide a microenvironment for GSC maintenance[4]. Both the germline and the somatic cyst stem cells divide asymmetrically: after division, one daughter remains in contact with the hub and self-renews while the other daughter is displaced away from the hub and initiates differentiation[5,6]. In the germline stem cell lineage, the differentiating daughter, termed the gonialblast, initiates four rounds of transit amplifying mitotic divisions with incomplete cytokinesis. The resulting 16 interconnected germ cells undergo premeiotic S phase in synchrony, become spermatocytes, and commit to terminal differentiation[7]. In the somatic cyst cell lineage, the differentiating daughter cell becomes a post-mitotic cyst cell, two of which enclose each gonialblast and its progeny, providing a supportive microenvironment necessary for the proper differentiation of the germ cells[8–10].

We identified a missense allele of *DREF* (*DREF*^*ts*^) that revealed a role for *DREF* in the maintenance of Drosophila male GSCs. *DREF* is known to function in cell growth, cell division, and DNA replication[11–13]. However, its role in these housekeeping processes has masked identification of other biological functions of *DREF*. The protein encoded by *DREF*^*ts*^ is able to function in cell division and cell survival but is defective for maintenance of GSCs in the testes. Analysis of mutant germline stem cells demonstrated defects in downstream targets of TGF-beta signaling. Genetic interactions with this allele of *DREF* suggested that DREF functions antagonistically to the chromatin regulators *Caf1/p55* and *Mi-2* to maintain GSCs. We propose that DREF may promote expression of self-renewal genes by overcoming transcriptional repression by a Mi-2 containing chromatin-remodeling complex.

## Results

### DREF function is required for male germline stem cell maintenance

**Fig 1:**
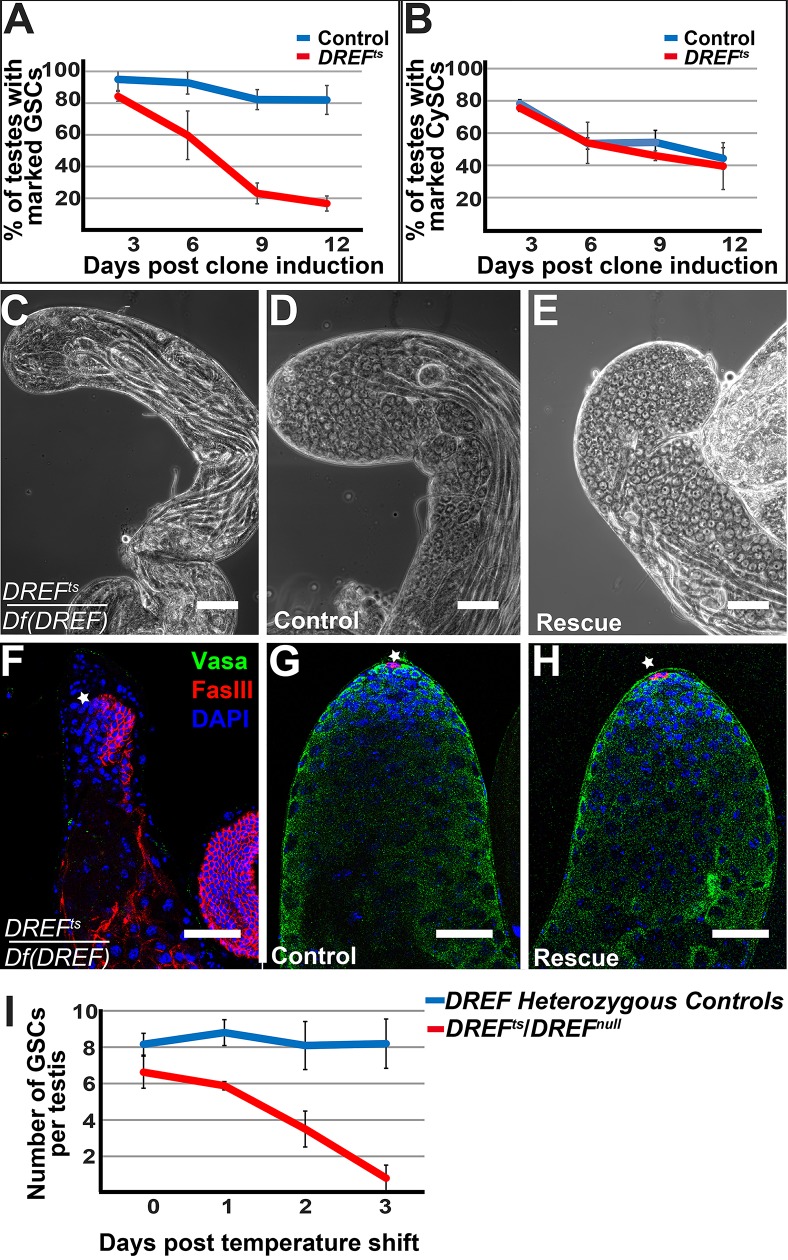
DREF function is required cell-autonomously for male GSC maintenance. (A, B) Percentage of testes with at least one marked DREF^ts^ mutant GSC (A, red), or DREF^ts^ mutant CySC (B, red) or marked control GSCs (A, blue) or control CySCs (B, blue) at the indicated time points post clone induction. (C-H) Phase contrast (C-E) and immunofluorescence (F-H) images of a *DREF*^*ts*^*/Df(DREF)* mutant testis (C, F), a sibling control testis (D, G), and a *DREF*^*ts*^*/Df(DREF)* testis expressing *UAS-DREF* under the control of the germ cell driver *nanos-Gal4VP16* (E, H). Apical tip of testis (denoted by the star) immuostained for vasa (green), FasIII (red), and DAPI (blue) is shown in (F-H). (I) Loss of *DREF*^*ts*^*/Df(DREF)* mutant GSCs grown at 22°C and then shifted to 30°C, compared with sibling controls. Scalebar: 50μm.

A temperature-sensitive allele of *DREF* (*DREF*^*ts*^) was discovered in an EMS-mutagenesis screen to identify genes required cell-autonomously for GSC maintenance in the Drosophila male germline. When GSCs homozygous for *DREF*^*ts*^ and negatively marked for GFP were generated using the FLP/FRT system [14] and grown at 25°C for 3 days post clone induction, 84.2±3.0% of the testes scored (n = 49) had at least one marked *DREF*^*ts*^ GSC adjacent to the hub. This is comparable to 95±3.0% of control testes (n = 38) with marked GSCs wild-type for *DREF* adjacent to the hub. However, the percentage of testes with at least one marked homozygous *DREF*^*ts*^ mutant GSC steadily decreased over time, so that by day 12 post clone induction, only 16.7±4.7% of testes (n = 91) contained one or more marked homozygous *DREF*^*ts*^ mutant GSCs, compared to 82.0±9.1% of testes (n = 64) in control flies (Fig 1A) (p<0.001). Surprisingly, *DREF*^*ts*^ did not have the same effect on CySC maintenance. The percentage of testes with marked CySCs in *DREF*^*ts*^/+ and wild-type controls were comparable at day 3 post clone induction (75.6% of *DREF*^*ts*^/+ (n = 38) to 78.3% (n = 49) of control testes) and also at day 12 post clone induction (39.2±14.5% of *DREF*^*ts*^/+ (n = 41) to 44.3±6.6% (n = 62) of control testes) (Fig 1B).

Complementation analysis indicated that the mutation responsible for the failure to maintain GSCs mapped to the *DREF* locus. The *DREF*^*ts*^ allele failed to complement *Df(2L)BSC17* [*Df(DREF)*, which deletes the *DREF* locus], as well as two independently-generated alleles, *DREF*^*KG09294*^ (referred to here as *DREF*^*null*^) and *DREF*^*NP4719*^ for early germ cell loss. Analysis of testes by phase contrast microscopy revealed that an average of 94.6± 5.6% testes (n = 58, p<0.0001) from newly eclosed *DREF*^*ts*^*/Df(DREF)* males grown at 25°C had late-stage germ cells (elongating spermatids) but lacked early germ cells (spermatogonia and primary spermatocytes), while 0% of testes from sibling *DREF* mutant/+ males lacked early germ cells (Fig 1C and 1D). The presence of elongating spermatids in testes from newly eclosed *DREF*^*ts*^*/Df(DREF)* males suggests that GSCs and early germ cells were initially present during development but were eventually lost due to differentiation. Immunofluorescence analysis of mutant testes confirmed the absence of GSCs and early germ cells; testes from newly eclosed *DREF*^*ts*^*/Df(DREF)* males grown at 25°C lacked Vasa-positive germ cells at the apical tip (Fig 1F), while testes from sibling controls had an abundance of Vasa-positive germ cells at the apical tip (Fig 1G). DREF function is also required for maintenance of female GSCs/early germ cells. In ovaries isolated from *DREF*^*ts*^*/Df(DREF*) mutant adult females grown at 25°C and examined three days after eclosion, 26.9% (n = 26) of ovarioles contained developing germ cell cysts and egg chambers but empty germaria (S1 Fig).

The loss of early germ cells in *DREF*^*ts*^*/Df(DREF)* males reared at 25°C was rescued by expression of a UAS-DREF cDNA transgene in early germ cells, confirming that the mutation causing GSC loss is in the *DREF* locus and that DREF function is required in germ cells for GSC maintenance. As assessed by phase contrast microscopy, only 5.37± 5.56% (n = 58) of testes from *DREF*^*ts*^*/Df(DREF)* males grown at 25°C contained early germ cells, while 100% (n = 57) of testes from *DREF*^*ts*^*/Df(DREF)* males expressing *UAS-DREF* cDNA under control of *nanos-Gal4-VP16* (Fig 1E) and 93.3 ± 8.80% (n = 59) of testes from *DREF*^*ts*^*/Df(DREF)* males expressing *UAS-DREF* cDNA under control of *Vasa-Gal4* (Fig 1E and Table 1) contained early germ cells. Immunofluorescence analysis revealed the presence of GSCs adjacent to the hub in *DREF*^*ts*^*/Df(DREF)* mutant testes expressing *UAS-DREF* cDNA under control of *nanos-Gal4-VP16* (Fig 1H).

**Table 1:** Rescue of early germ cell loss in DREF mutants by different transgenes.

Genotype	Average Percentage of testes with early germ cells	SD	P-value
DREF^ts^, UAS-DREF/Df(2L)BSC17	4	4.87	
DREF^ts^, UAS-DREF/Df(2L)BSC17; nanos-Gal4VP16/+	100	0	1.05E-10
DREF^ts^, UAS-DREF/Df(2L)BSC17; Vasa-Gal4/+	91.67	11.78	9.44E-08
DREF^ts^/Df(2L)BSC17; UAS-E-Cadherin/nanos-Gal4VP16	2.67	3.37	0.77

The GSC loss phenotype of *DREF*^*ts*^*/DREF*^*null*^ flies was temperature-dependent. As assayed by phase contrast microscopy, 100 ± 0% (n = 82) of testes isolated from *DREF*^*ts*^*/DREF*^*null*^ transheterozygous flies grown at 22°C had early germ cells present on the day of eclosion. However, when *DREF*^*ts*^*/DREF*^*null*^ flies were grown at 25°C, only 20.2 ± 12.9% (n = 375) of testes scored contained early germ cells on the day of eclosion. *DREF*^*ts*^*/DREF*^*null*^ flies grown at 30°C failed to survive to adulthood and died in late third instar larval and/or pupal stages (Table 2).

**Table 2:** Viability of DREF mutants and different temperatures.

Genotype	22°C	25°C	30°C
DREF^ts^/DREF^ts^	Viable	Viable, early germ cell loss (~25%)	lethal (3rd instar-pupal stages)
DREF^ts^/DREF^KG09294^	Viable	Viable, early germ cell loss (~75%)	lethal (3rd instar-pupal stages)
DREF^ts^/DREF^NP4719^	viable	Viable, early germ cell loss (~80%)	lethal (3rd instar-pupal stages)
DREF^ts^/Df(2L)BSC17	viable	Viable, early germ cell loss (~95%)	lethal (3rd instar-pupal stages)
DREF^KG09294^/DREF^KG09294^	lethal (embryonic/first instar)	lethal (embryonic/first instar)	lethal (embryonic/first instar)
DREF^KG09294^/DREF^NP4719^	lethal (embryonic/first instar)	lethal (embryonic/first instar)	lethal (embryonic/first instar)
DREF^NP4719^/DREF^NP4719^	lethal (embryonic/first instar)	lethal (embryonic/first instar)	lethal (embryonic/first instar)
DREF^NP4719^/Df(2L)BSC17	lethal (embryonic/first instar)	lethal (embryonic/first instar)	lethal (embryonic/first instar)
DREF^KG09294^/Df(2L)BSC17	lethal (embryonic/first instar)	lethal (embryonic/first instar)	lethal (embryonic/first instar)
Df(2L)BSC17/Df(2L)BSC17	lethal (embryonic)	lethal (embryonic)	lethal (embryonic)

Sequencing of the DREF coding region revealed that the *DREF*^*ts*^ allele had two amino acid substitutions (Methionine 651 to Leucine and Glycine 652 to Alanine), which occur in a domain that has been shown to be responsible for co-factor binding in Drosophila DREF[15,16]. Germ cells homozygous mutant for *DREF*^*ts*^ still expressed DREF protein as assayed by immunostaining 3 days and 6 days post clone induction. In contrast, at day 3 post clone induction, DREF protein expression was not detected in DREF^null^ mutant germ cells by immunofluorescence using anti-DREF antibodies (S2 Fig). Furthermore, GSCs in testes from *DREF*^*ts*^*/DREF*^*nul*l^ versus *DREF*^*null*^/+ flies grown at 22°C until eclosion then shifted to 30°C for 2 days failed to show significantly different levels of DREF protein (0.63±0.15 n = 18 GSCs versus 0.72±0.21 n = 24 GSCs, Materials and Methods).

### *DREF*^*ts*^ cells proliferate and are able to differentiate

**Fig 2:**
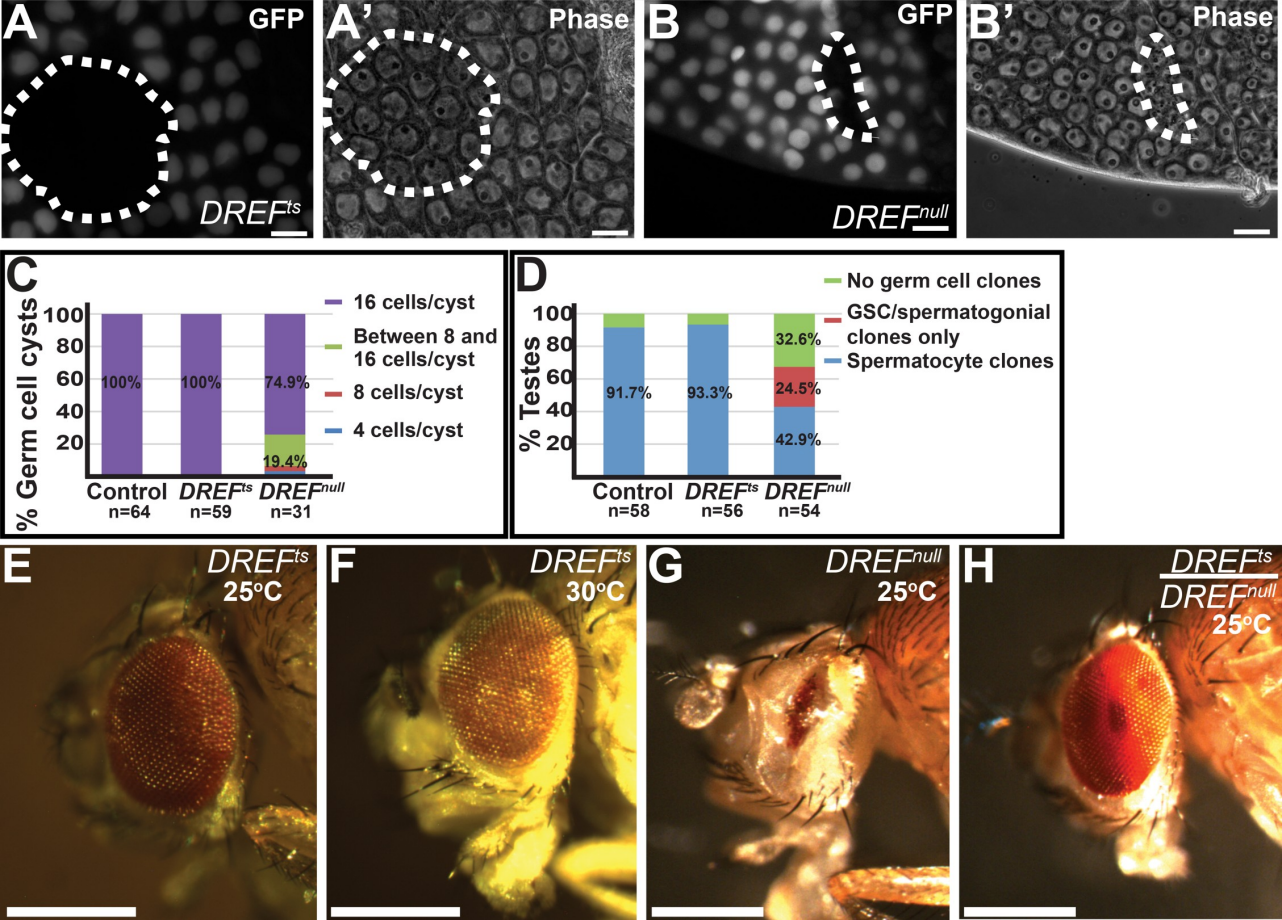
*DREF*^*ts*^ does not affect germ cell differentiation in the testis and cell proliferation in the testis and eye. (A-B) *DREF*^*ts*^ mutant spermatocyte clones (dashed-outline in A, A’) are comparable in size to neighboring spermatocytes with wild-type DREF, while *DREF*^*null*^ mutant spermatocyte clones (dashed-outline B, B’) show a significant reduction in cell size compared to neighboring wild-type spermatocytes. (C) *DREF*^*ts*^ and control spermatocytes contain the proper number of germ cells per cyst (16) whereas *DREF*^*null*^ mutant clones have cysts containing less than 16 cells. (D) Percentage of testes that contain marked spermatocyte clones (blue), marked early germ cell clones (red), or neither (green). (E-G) Analysis of the role of *DREF* in eye tissue using the EGUF/hid method to induce clones. *DREF*^*ts*^ mutant clones induced in the eye appear comparable to wild type whether grown at 25°C (E) or 30°C (F), while *DREF*^*null*^ clones cause cell death and display smaller eyes compared to controls (G). (H) Eyes from *DREF*^*ts*^*/DREF*^*null*^ transheterozygous flies grown at 25°C also have a wildtype appearance. Scalebar: 10 μm for (A and B).

The *DREF*^*ts*^ allele allowed separation of the role of *DREF* in stem cell maintenance from a general role of *DREF* in cell survival and proliferation. Male GSCs homozygous mutant for the *DREF*^*ts*^ allele appeared to be lost because they differentiate more often than self-renew. While homozygous *DREF*^*ts*^ mutant GSCs were rapidly lost from the apical hub region of the testis at 25°C, their marked clonal progeny appeared to differentiate normally to at least the spermatocyte stage, as assessed by phase contrast microscopy. Cysts of spermatocyte clones homozygous for the DREF^ts^ allele examined on day 4 post clone induction contained 16 germ cells per spermatocyte cyst, as expected after 4 rounds of transit amplification divisions, and were comparable in size to neighboring wild-type spermatocytes (Fig 2A, 2A’ and 2C). In contrast, loss of DREF function in germline clones homozygous for the *DREF*^*null*^ allele or due to expression in early gem cells of an RNAi hairpin directed against DREF mRNA under control of *nanos-Gal4-VP16* resulted in defects in germ cell survival, growth, proliferation, and/or differentiation. At day 4 post induction of marked clones homozygous for DREF^null^, 32.6% of the testes scored contained no *DREF*^*null*^ clones, 24.5% had marked GSCs and/or spermatogonia but no late spermatocyte cysts homozygous for *DREF*^*null*^, and only 42.9% contained cysts with recognizable spermatocytes at 25°C (Fig 2D). The cysts of *DREF*^*null*^*/DREF*^*null*^ mutant spermatocytes in the clones appeared smaller in size compared to neighboring *DREF*^*null*^*/*+ germ cell cysts (Fig 2B and 2B’). In addition, about 25% of the germ cell cysts that made it to the spermatocyte stage exhibited fewer than 16 cells per cyst (Fig 2C). Together, these observations suggest that lack of DREF function may cause developing germ cell cysts to either die, grow slowly, or fail to initiate the differentiation program. Similarly, knockdown of DREF function in late spermatogonia and spermatocytes by expressing *UAS-DREF-RNAi* under the control of a *Bam-Gal4* driver resulted in extensive cell death and absence of meiotic and post-meiotic stages (S3 Fig).

Consistent with the finding that homozygous *DREF*^*ts*^ germ cells proliferate and differentiate normally, cells homozygous for *DREF*^*ts*^ in the eye were able to proliferate and differentiate, unlike cells homozygous for *DREF*^*null*^. Flies with eyes entirely composed of cells homozygous mutant for *DREF*^*null*^ generated using the EGUF-hid method [17] and reared at 25°C had very small eyes (Fig 2G). In contrast, eyes entirely homozygous mutant for *DREF*^*ts*^ appeared similar in size and morphology to wild type controls, whether grown at 25°C (Fig 2E) or 30°C (Fig 2F). Additionally, eyes in *DREF*^*ts*^*/ DREF*^*null*^ transheterozygous flies grown at 25°C were wild type in size and appearance (Fig 2H).

The temperature-sensitivity of the *DREF*^*ts*^ allele allowed analysis of how GSCs are lost when shifted from permissive to non-permissive temperature. GSCs present when *DREF*^*ts*^*/DREF*^*null*^ males were grown at 22°C until eclosion were lost within 2–3 days upon shifting the males to 30°C (Fig 1I). On the day of eclosion, *DREF*^*ts*^*/DREF*^*null*^ males grown at 22°C had an average of 6.6± 0.9 GSCs around the hub (n = 22 testes), compared to 8.2± 0.6 GSCs in heterozygous control testes (n = 27 testes). In contrast, testes from *DREF*^*ts*^*/DREF*^*null*^ males grown at 22°C then shifted to 30°C for 3 days after eclosion had an average of 0.8± 0.7 GSCs (n = 69 testes), while sibling control testes from DREF mutant/+ males had an average of 8.19±1.4 GSCs per testis (n = 59 testes) (p<0.0001) (Fig 1I).

**Fig 3:**
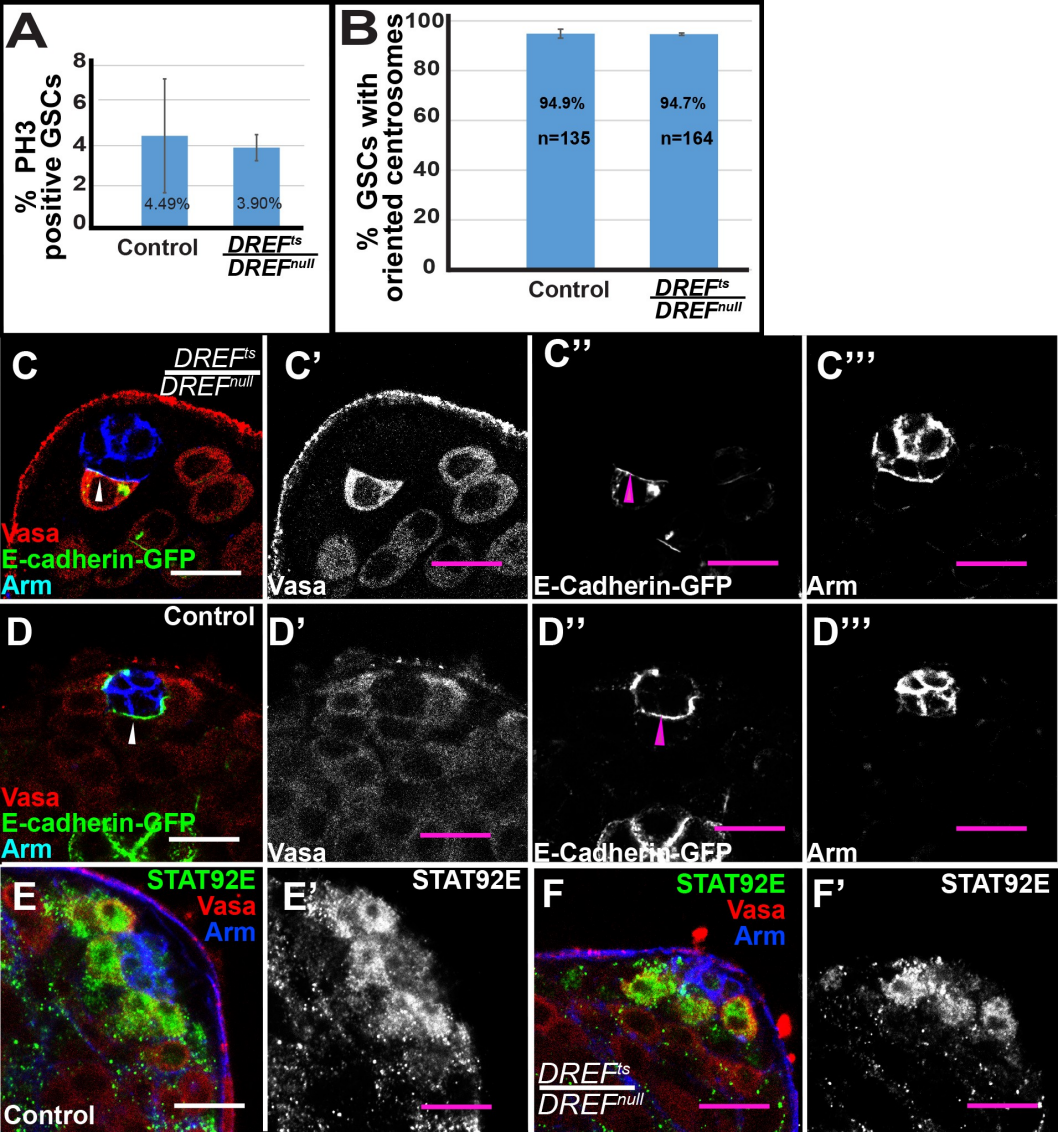
*DREF*^*ts*^*/DREF*^*null*^ GSCs do not show defects in adhesion or STAT signaling. (A) Percentage of GSCs in mitosis in DREF^ts^/DREF^null^ mutant and sibling control testes at 2 days post-temperature shift. (B) Percentage of oriented centrosomes in heterozygous control and DREF^ts^/DREF^null^ GSCs 2 days post shift to 30°C. (C, D) E-Cadherin-GFP localizes to the hub-GSC interface (arrowhead) in both *DREF*^*ts*^*/DREF*^*null*^ mutant testes (C-C’’’) and sibling control testes (D-D’’’). (E, F) STAT protein is expressed comparably in both heterozygous control GSCs (E-E’) and *DREF*^*ts*^*/DREF*^*null*^ mutant GSCs (F-F’). Scalebar: 10μm.

The loss of *DREF* function when *DREF*^*ts*^*/DREF*^*null*^ males were shifted to 30°C did not appear to affect the rate of GSC division or survival. The percentage of GSCs in mitosis scored by immunostaining for phosphorylated-Threonine 3 of histone H3 (PH3) was similar in testes isolated from *DREF*^*ts*^*/ DREF*^*null*^ transheterozygotes (3.90 ± 0.65%, n = 154 GSCs) and sibling control flies (4.49 ±2.84%, n = 178 GSCs) that were grown at 22°C, shifted to 30°C at eclosion and then held at 30°C for two days (Fig 3A). Additionally, TUNEL assays failed to detect dying GSCs in testes from either *DREF*^*ts*^*/ DREF*^*null*^ transheterozygotes or control flies (n = 83 and n = 94 GSCs, respectively), while in both cases some dying spermatogonial cysts were detected.

The loss of GSCs in *DREF*^*ts*^*/ DREF*^*null*^ testes did not appear to be due to loss of hub-GSC adhesion. Expression of *UAS-E-Cadherin-GFP* specifically in early germ cells using the *nanos-Gal4-VP16* driver resulted in localization of E-Cadherin-GFP protein to the hub-GSC interface in both *DREF*^*ts*^/ *DREF*^*null*^ (Fig 3C–3C’’’) and *DREF*^*ts*^/+ (Fig 3D–3D’’’) GSCs in testes isolated from males grown at 22°C until eclosion then shifted to 30°C for two days. Expression of E-Cadherin-GFP using the UAS-GAL4 system in early germ cells did not rescue the loss of GSCs in *DREF*^*ts*^/ *DREF*^*null*^ temperature-shifted flies, as testes from *DREF*^*ts*^*/ DREF*^*null*^ males shifted to 30°C for two days contained similar numbers of GSCs per testis whether or not they expressed *UAS-E-Cadherin-GFP* under control of *nanos-Gal4-VP16* (average of 0.9±0.2 GSCs per testis (n = 52 testes) and 0.8±0.7 GSCs per testis (n = 59 testes), respectively).

*DREF*^*ts*^*/ DREF*^*null*^ germ cells adjacent to the hub also oriented their centrosomes as in wild-type GSCs, where one centrosome is positioned adjacent to the hub-GSC interface throughout the cell cycle, resulting in oriented GSC division[5]. In testes isolated from *DREF*^*ts*^*/ DREF*^*null*^ males grown at 22°C then shifted to 30°C for two days after eclosion, an average of 92.78 ± 3.76% (n = 201 from 62 testes) of GSCs that remained next to the hub and contained two centrosomes had one centrosome adjacent to the hub-GSC interface, similar to control *DREF*^*ts*^/+ GSCs (94.1 ± 1.07%, n = 239 GSCs from 30 testes) (Fig 3B).

Hub-GSC adhesion and oriented centrosome positioning are two features of GSCs that depend on activation of the JAK-STAT pathway in response to Unpaired (Upd) ligand secreted from the hub[10,18]. Consistent with intact hub-GSC attachment and correct positioning of centrosomes, GSCs in testes from *DREF*^*ts*^*/ DREF*^*null*^ males grown at 22°C then shifted to 30°C for two days post-eclosion expressed STAT92E protein, a downstream target of JAK-STAT signaling, at levels comparable to sibling control GSCs (Fig 3E–3E’ and 3F–3F’).

### Impaired TGF-beta signal transduction pathway in *DREF*^*ts*^*/DREF*^*null*^ GSCs

**Fig 4:**
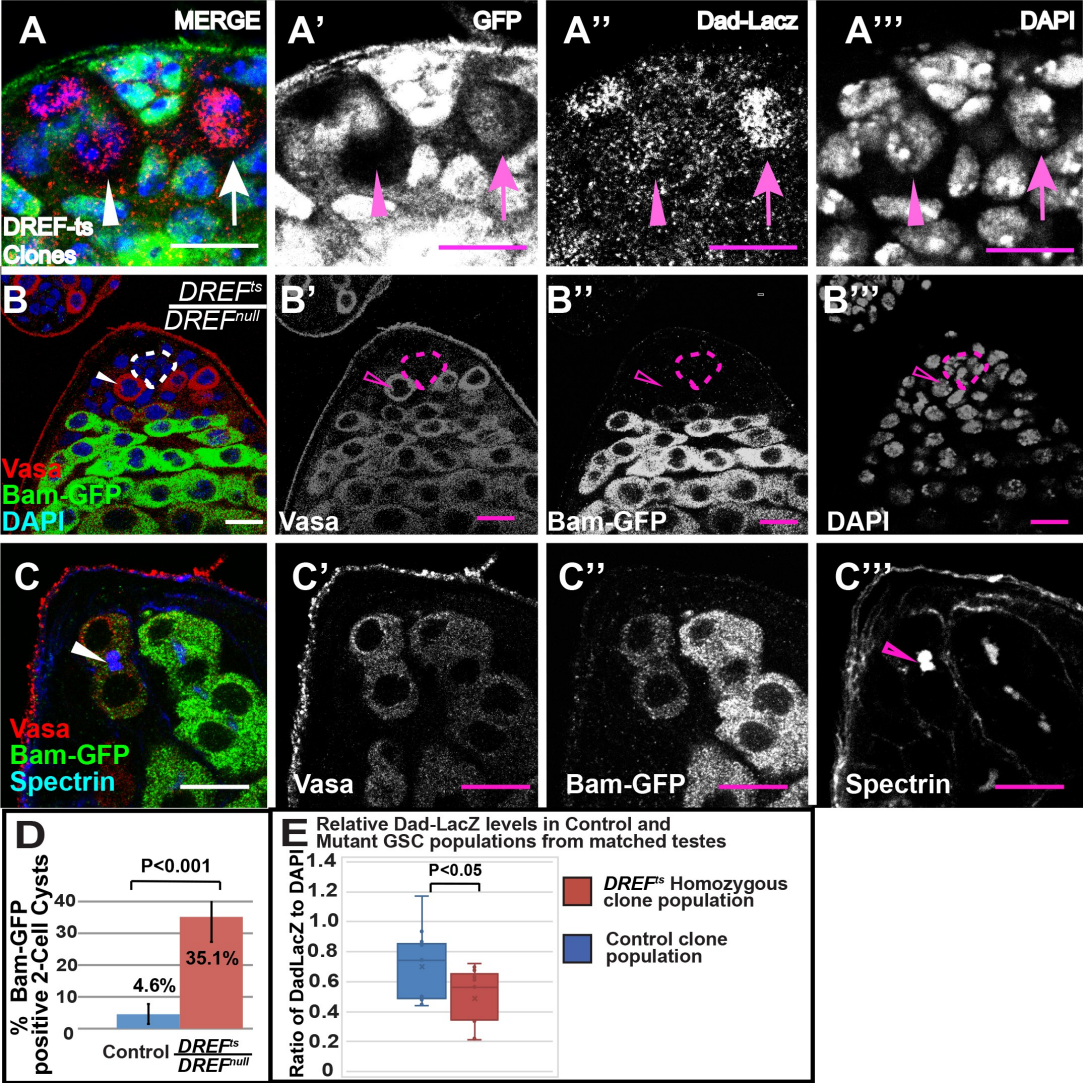
Impaired TGF-beta signal transduction pathway in *DREF*^*ts*^*/DREF*^*null*^ GSCs. (A-A’’’) DREF^ts^ homozygous mutant GSCs (arrowhead) expressed reduced levels of the TGF-beta reporter *Dad-LacZ* compared with neighboring heterozygous controls (arrow). (B-B’’’) *DREF*^*ts*^*/DREF*^*null*^ mutant GSCs (B, arrowhead) did not express the differentiation marker Bam. (C) *DREF*^*ts*^*/DREF*^*null*^ two-cell germline cysts frequently expressed Bam. (D) Quantification of percentage of two-cell germline cysts expressing Bam in both sibling control and *DREF*^*ts*^*/DREF*^*null*^ mutant testes. (E) Whisker and Box plot of average ratio of Dad-LacZ to DAPI in Mutant and Control GSC populations from testes that contain both heterozygous control and marked homozygous mutant clones in the same testis. Scalebar: 10μm.

Many GSCs homozygous mutant for *DREF*^*ts*^ showed reduced expression of a reporter of TGF-beta signaling, *Dad-LacZ*, compared to their *DREF*^*ts*^*/+* neighbors next to the hub. In wildtype, *Dad-LacZ* is primarily expressed in GSCs, the gonialblast, and in later stage somatic cyst cells[19]. In many germ cells next to the hub made homozygous for *DREF*^*ts*^ by FLP induced mitotic recombination, LacZ staining was often drastically reduced compared to neighboring *DREF*^*ts*^/*+* GSCs (Fig 4A–4A’’’). The effect was variable, however, with some GSCs homozygous for *DREF*^*ts*^ appearing to have normal levels of LacZ, likely leading to the observed gradual loss of *DREF*^*ts*^ mutant GSCs. As a population, mutant GSCs showed a 30.3% reduction (P<0.05, n = 14 GSCs in 11 testes, Fig 4E) in Dad-LacZ staining intensity relative to control GSCs (n = 17 GSCs in 11 testes) within the same testes as quantified by ImageJ (Materials and Methods).

Consistent with a reduced response to TGF-beta signaling, the differentiation marker Bag of Marbles (Bam) was expressed earlier than normal in germ cells in *DREF*^*ts*^*/DREF*^*null*^ testes. Bam protein is normally detected by antibody staining in 4- to 16-cell spermatogonial cysts[20]. Likewise, using a reporter line driving GFP-tagged Bam protein expressed from its own promoter and regulatory elements[21], Bam-GFP expression, as detected by anti-GFP immunostaining, was first detected in 4 cell cysts, with very few 2 cell cysts scoring positive. GSCs from *DREF*^*ts*^*/ DREF*^*null*^ males grown at 22°C until eclosion then shifted to 30°C for two days did not express Bam-GFP in GSCs adjacent to the hub (n = 36 GSCs from 14 testes), similar to GSCs from sibling controls that were either *DREF*^*ts*^*/+* or *DREF*^*null*^ */+* (n = 100 GSCs from 12 testes) (Fig 4B–4B’’’). Likewise, gonialblasts, defined as single germ cells away from the hub containing a dot-fusome, from *DREF*^*ts*^*/DREF*^*null*^ temperature shifted males also did not express Bam-GFP (n = 23 gonialblasts from 14 testes), similar to the gonialblasts in heterozygous controls, in which Bam-GFP was not detected (n = 31 gonialblasts from 12 testes). However, there was a marked increase in the percentage of Bam-GFP positive two-cell cysts in *DREF*^*ts*^*/DREF*^*null*^ mutant testes, where 35.1% of the two-cell cysts analyzed (n = 37 counted in 14 testes) were positive for Bam-GFP, compared with only 4.6% of two-cell cysts scored as positive for Bam-GFP in testes from *DREF*^*ts*^*/+* or *DREF*^*null*^ */+* sibling controls (n = 44 cysts counted in 12 testes)(Fig 4C’–4C’’’ and 4D).

### Forced overactivation of the JAK-STAT signaling pathway did not rescue loss of GSCs in *DREF*^*ts*^ mutants

**Fig 5:**
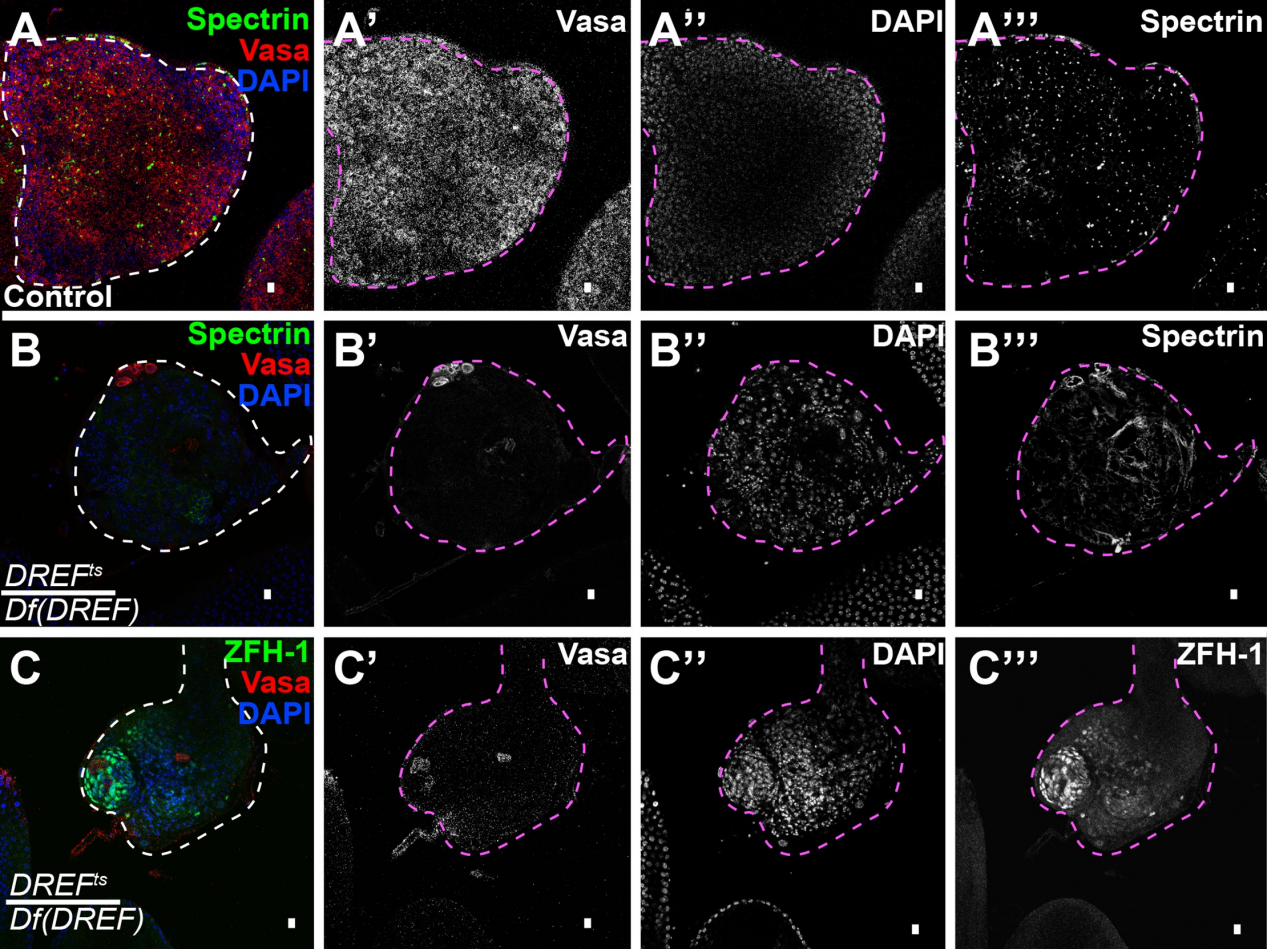
DREF is required independently of the JAK-STAT pathway. (A, B, C) Ectopic expression of *Upd* in the germline leads to an overproliferation of GSC-like cells in heterozygous control testes (A-A’’’), but not in *DREF*^*ts*^*/Df(DREF)* testes (B-B’’’). *DREF*^*ts*^*/Df(DREF)* testes ectopically expressing *Upd* in the germline still contain an overabundance of Zfh-1 positive CySC-like cells (C-C’’’). Scalebar: 10μm.

Function of *DREF* appeared to be required for maintenance of germline stem cell state even under the condition of forced ectopic expression of the ligand Upd, which activates the JAK-STAT signal transduction pathway. In control *DREF*^*ts*^/+ or *Df(DREF)*/+ flies, ectopic expression of *UAS-Upd* in early germ cells under the control of *nanos-Gal4-VP16* at 25°C resulted in 100% (n = 68 testes) of testes examined containing an overabundance of GSC-like Vasa-positive cells with dot spectrosomes, as well as many CySC-like cells positive for Zfh-1 (Fig 5A–5A’’’). Under the same conditions, in contrast, only 4.98±5.64% (n = 66 testes) of *DREF*^*ts*^*/Df(DREF)* testes ectopically expressing *UAS-Upd* under control of *nanos-Gal4-VP16* exhibited an overabundance of GSC and CySC-like cells. Rather, the vast majority of testes (95.02%, n = 66) from *DREF*^*ts*^*/Df(DREF)* males carrying *nanos-Gal4-VP16; UAS-Upd* grown at 25°C had an abundance of CySC-like cells but few or no germ cells as assayed by immunostaining for Vasa (Fig 5B–5B’’’). The abundance of Zfh-1 positive CySC-like cells in many of the *DREF*^*ts*^*/Df(DREF)* testes suggested that sufficient Upd was expressed early, prior to the GSC loss (Fig 5C–5C’’’).

### Caf1 and Mi-2 genetically antagonize DREF to inhibit GSC Self-Renewal

**Fig 6:**
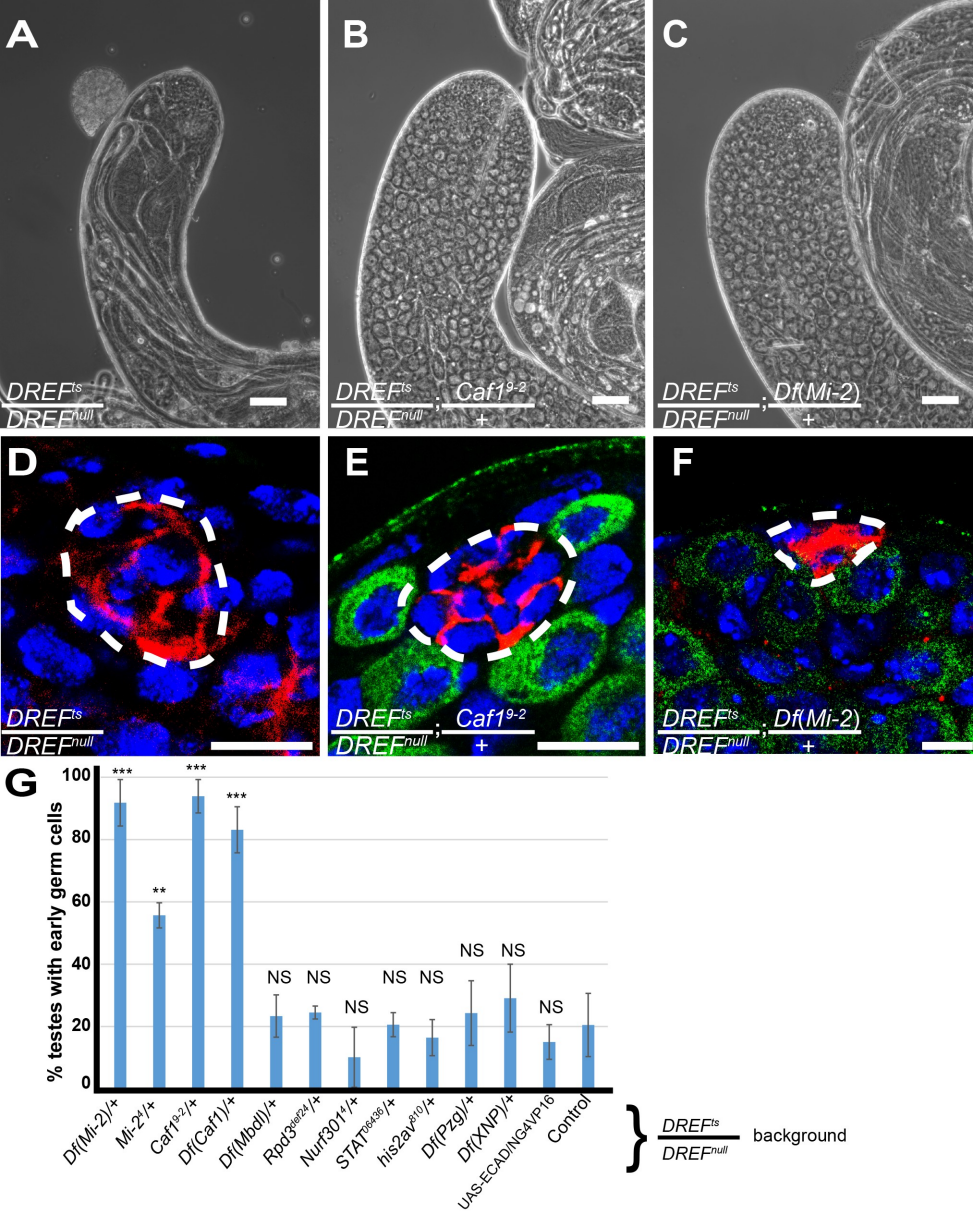
Mi-2 and Caf1 genetically antagonize DREF. (A-F) Phase contrast images and immunostaining images of (A, D) *DREF*^*ts*^*/DREF*^*null*^ testes; (B, E) *DREF*^*ts*^*/DREF*^*null*^ *; Caf*^*9-2*^*/+* testes, and (C, F) *DREF*^*ts*^*/DREF*^*null*^ *; Df(Mi-2)/+* testes, respectively. Half-dose reduction of *Caf1* and *Mi-2* resulted in restoration of early germ cells as assessed by phase contrast microscopy (A-C) and germline stem cells as assessed by immunostaining (D-F). (G) Percentage of testes with early germ cells as assessed by phase contrast microscopy upon half-dosage reduction of gene function in a *DREF*^*ts*^*/DREF*^*null*^ mutant background. Scalebar: 50μm (A-C) and 10μm (*D-F*).

The *DREF*^*ts*^ allele provided a sensitized background in which to screen for genetic interactors important for male GSC differentiation vs. maintenance. When *DREF*^*ts*^*/ DREF*^*null*^ flies were raised at 25°C, 79.8±12.9% of testes (n = 375) from newly eclosed males had few or no early germ cells and displayed elongating spermatid bundles close to the apical tip (Fig 6A). Strikingly, reducing the gene dosage of either *Chromatin assembly factor 1*, *p55 subunit* (*Caf-1/p55*), a subunit of multiple chromatin-modifying complexes including the NuRD complex, or *Mi-2*, a subunit of the Nucleosome Remodeling and Deacetylase (NuRD) complex and the dMEC complex, rescued the *DREF* early germ cell loss phenotype. While only 20.2±12.9% (n = 375) of testes from newly eclosed *DREF*^*ts*^*/DREF*^*null*^ males had early germ cells (Fig 6A), 93.9±5.35% (n = 62) of testes from *DREF*^*ts*^*/DREF*^*null*^*; Caf1*^*9-2*^*/+* males contained both plentiful early germ cells and abundant spermatocytes as assessed by phase contrast microscopy (Fig 6B). Similarly, 91.8±7.5% (n = 76) of testes from *DREF*^*ts*^*/DREF*^*null*^*; Df(Mi-2)/+* and 55.6±4.0% of testes from *DREF*^*ts*^*/DREF*^*null*^*; Mi-2*^*4*^*/+* males contained many early germ cells and spermatocytes (Fig 6C). Immunofluorescence analysis revealed that reducing *Caf1* or *Mi-2* dosage by half in a *DREF*^*ts*^*/DREF*^*null*^ background restored the presence of GSCs next to the hub (Fig 6D–6F). The suppression of the *DREF*^*ts*^*/DREF*^*null*^ GSC loss phenotype by lowering the dosage of either *Mi-2* or *Caf1*, both of which are components of the NuRD complex, suggests that DREF and the NuRD complex may act antagonistically to influence GSC maintenance. However, lowering the dose of *Rpd3* or *Mbd-like*, two other subunits of the NuRD complex, did not suppress the early germ cell loss phenotype in DREF mutant testes as assessed by phase contrast microscopy (Fig 6G). Similarly, lowering the dose of *XNP*, a member of the DREF-containing XNP/dATRX repression complex, or *Putzig*, a member of the DREF-TRF2 complex, did not affect the germ cell loss phenotype, with only 29.1±10.9% (n = 74) and 23.3±10.4% (n = 41), respectively, of testes scored by phase contrast microscopy containing visible early germ cells (Fig 6G). *TRF2*, which is male lethal, could not be tested in the same manner.

### Mi-2 and Caf1 are required for GSC maintenance

**Fig 7:**
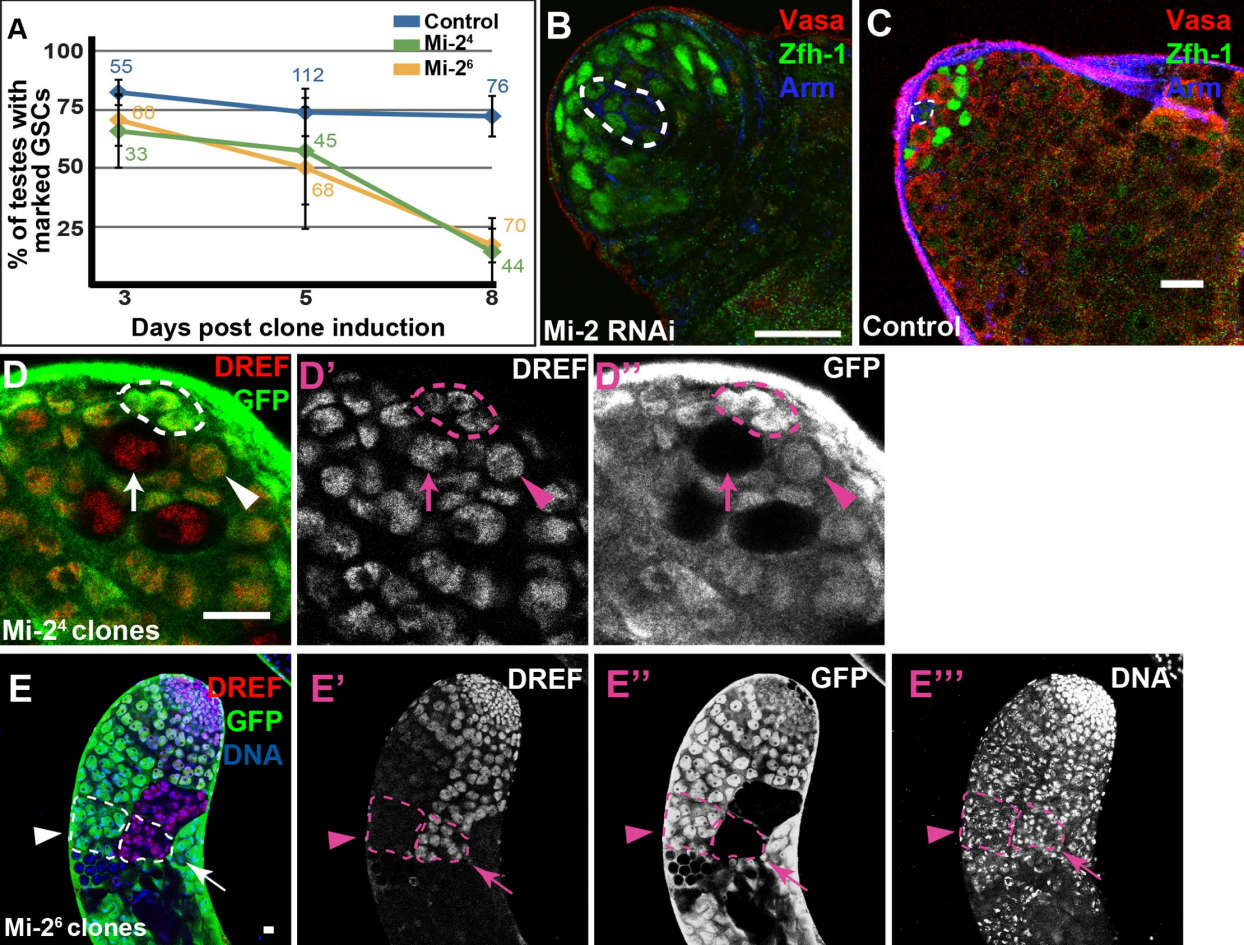
Mi-2 is required autonomously for GSC maintenance. (A) Percentage of testes containing at least one marked *Mi-2* mutant GSC or marked control GSC at different days after clone induction. Labels on the graph are the number of testes counted at sample time point. (B, C) Expression in the germline of RNAi directed against *Mi-2* lead to loss of GSCs and early germ cells (B) compared to control testes (C) as assessed by immunostaining with antibodies against Vasa (red) to detect germ cells, Zfh-1 (green) to detect CySCs and Armadillo (blue) to detect the hub. (D) DREF protein levels did not appear to alter upon loss of Mi-2 function as assessed by immunostaining in *Mi-2*^*4*^ mutant GSCs (white arrow) compared to neighboring heterozygous GSC (yellow arrow) 4 days after clonal induction. Dotted line in B-D marks the hub (E) *Mi-2*^*6*^ mutant spermatocyte clones (arrow) show higher levels of DREF protein compared to neighboring, temporally-controlled heterozygous spermatocyte clones (arrowhead). Scalebar: 15μm (A and B) 10μm (D and E).

*Mi-2* is required cell-autonomously for GSC maintenance in the testis. GSCs made homozygous mutant for either the *Mi-2*^*4*^ (frameshift) or the *Mi-2*^*6*^ (premature stop codon) allele using the Flp-FRT system were lost over time. At day 3 post clone induction, 65.9±16.1% of testes scored (n = 33) for *Mi-2*^*4*^ and 70.7±11.5% of testes scored (n = 66) for *Mi-2*^*6*^ contained marked homozygous mutant GSCs, compared to control clones, for which 82.5±5.9% of testes scored (n = 55) contained marked GSCs. By day 8 post clone induction, however, only 14.3±15.2% (n = 44) for *Mi-2*^*4*^ and 17.2±7.9% (n = 70) for Mi-2^6^ of testes scored contained marked homozygous mutant GSCs, while the percentage of control testes with marked GSCs was significantly higher (72.3±9.1%, n = 76, p<0.001) (Fig 7A). Germ cells homozygous mutant for *Mi-2* were able to differentiate into spermatocytes. Expression of RNAi directed against *Mi-2* using the *nanos-Gal4-VP16* driver also resulted in early germ cell loss, confirming a role for *Mi-2* in GSC maintenance (Fig 7B and 7C). Similarly, RNAi targeting *Caf1* expressed using the *nanos-Gal4-VP16* driver also resulted in GSC loss, demonstrating a requirement for *Caf1* for GSC maintenance (S4 Fig). Homozygous *Mi-2*^*4*^ or *Mi-2*^*6*^ mutant GSCs remaining at day 5 post clone induction expressed DREF protein at levels comparable to neighboring *Mi-2/+* heterozygous GSCs, as detected by immunostaining (Fig 7D). However, later stage germ cells lacking *Mi-2* function demonstrated prolonged DREF expression, visible as persistent high levels of DREF protein in spermatocyte cysts in germline clones homozygous mutant for *Mi-2*, compared to neighboring *Mi-2/+* spermatocytes (Fig 7E–7E’’’).

## Discussion

### The *DREF*^*ts*^ allele demonstrates a specific requirement for DREF in adult stem cell self-renewal

Drosophila DREF was initially isolated based on its ability to bind the DNA-Replication Related Element (DRE), an 8bp sequence 5’TATCGATA’3 located upstream of many genes related to DNA replication. Binding of DREF protein to the DRE has been shown to activate expression of genes regulating cell division, including *DNA polymerase*, *E2F*, and *Cyclin A*[22–24]. In addition, Drosophila DREF has been shown to act downstream of the TOR [13] signaling pathway and Drosophila DREF and its human homolog, hDREF, have also been shown to control cell growth by regulating the expression of ribosomal genes and *histone H*1[13,25,26]. The DRE bound by DREF is known to be a key *cis*-regulatory component of a class of core promoters different from the canonical TATA box containing promoters[27]. Binding of DREF protein to the DRE recruits TRF2, a transcription factor related to TATA-box-binding protein (TBP), directing recognition of these alternate core promoters, regulating, for example expression of the *proliferating cell nuclear antigen* (*PCNA*)[28]. DREF Protein binds to and potentially regulates 1,961 distinct loci in the genome[29]. For example, DREF has been shown to regulate chromatin by: 1) by activating transcriptional expression of chromatin regulators such as *brahma*, *moira* and *osa*[22,30], *Mes4*[31], and *HP6*[32], 2) physically interacting with XNP/dATRX and potentially targeting them to regions in the genome[16], 3) competing with the chromatin insulator Boundary Element Association Factor 32 (BEAF32) for a mutual binding site[29,33], and 4) regulating the HET-A, TART, TAHRE array (HTT) array in Drosophila[34]. Previous reports, and our work here, showed that null alleles of *DREF* have defects in cell division and cell growth in tissues ranging from the eye[12,22], salivary glands[12], the imaginal wing discs[35], and the testis (this report).

The *DREF*^*ts*^ allele we identified a role for *DREF* in self-renewal of germ line stem cells, genetically separable from the previously-defined role of *DREF* in cell proliferation. The *DREF*^*ts*^ allele did not strongly affect cell viability and division in the eye, and somatic cyst stem cells (CySCs) homozygous mutant for the *DREF*^*ts*^ allele were able to self-renew and differentiate normally. However, in flies homozygous mutant for the *DREF*^*ts*^ allele, GSCs were not maintained, although the progeny of mutant GSCs were able to differentiate into spermatocytes. It is possible that the *DREF*^*ts*^ allele might affect specific physical interactions between DREF and particular binding partner(s) required in GSCs but not in many other cell types, resulting in stem cell loss.

Intriguingly, previous research suggests that the domain mutated in *DREF*^*ts*^ (the CR3 domain) does not contribute to the DNA binding or dimerization functions of DREF, but may play a role in the ability of DREF to bind and interact with different cofactors[36]. Overexpression of the CR3 domain of DREF has been shown to have a dominant-negative effect on DREF function, possibly by competing for normal binding partners of endogenous DREF[36]. In support of this view, DREF has been shown to bind to the chromatin remodeling factor XNP through its CR3 domain[16]. When bound to XNP, DREF can function as a transcriptional repressor, in contrast to its typical role as a transcriptional activator when bound to TRF2[16]. However, *XNP* showed no genetic interaction with the *DREF*^*ts*^*/DREF*^*null*^ mutant phenotype, suggesting that either *XNP* is not dosage sensitive or that the role of DREF in GSC maintenance is not mediated through the DREF-XNP complex.

Although an RNAi screen by *Yan et al*. [37] showed a requirement for *DREF* in the female germline, it is important to note that in our hands complete loss of function of *DREF* (through either RNAi or null alleles) causes severe phenotypes likely due to the role of DREF in housekeeping functions required for cell growth and division, which parallels the multiple, complex defects *Yan et al*. [37] notes in knockdown of DREF in the female germline.

### DREF is required for GSC maintenance independently of the JAK-STAT signaling pathway

The JAK-STAT signaling pathway plays pivotal roles in regulating the two adult stem cell populations in the testis. In male GSCs, the JAK-STAT signal transduction pathway is required cell autonomously for adhesion to the hub and oriented divisions, but not for self-renewal[18]. Many mutants that result in GSC loss, such as *NURF301* [38] have reduced JAK-STAT signaling, possibly resulting in loss of GSC adhesion to the hub and subsequent differentiation. In contrast, D*REF*^*ts*^*/DREF*^*null*^ GSCs had normal levels of STAT protein and did not appear to be defective in hub-GSC adhesion, as evidenced by localization of E-Cadherin and proper centrosome orientation, suggesting that they are not likely lost due to defects in JAK-STAT signaling.

JAK-STAT activation is also required in CySCs for self-renewal[18,39]. Forced activation of JAK-STAT signaling in the testes, either by expressing constitutively active JAK in CySCs or by forced ectopic expression of the activating ligand Upd in germ cells, results in an apparent failure of CySCs to differentiate[18]. As a consequence of the early CySC-like state, the neighboring germ cells fail to differentiate and the testis is filled with GSC-like and CySC-like cells. This overproliferation of GSC-like cells due to forced activation of the JAK-STAT pathway can mask or override the GSC-loss phenotype in *his2Av* or *GEF26* mutants[40,41]. Although, like *his2Av* and *GEF26* mutant GSCs, *DREF*^*ts*^ GSCs are lost to differentiation, the outcome of combining *DREF*^*ts*^ with forced expression of Upd ligand in germ cells was strikingly different, with the *DREF*^*ts*^/*Df(DREF)* germ line stem cell loss phenotype predominating even with forced activation of JAK-STAT signaling due to ectopic expression of Upd. Thus, while *his2av* and *GEF26* may be important for fine-tuning the balance between self-renewal and differentiation, the function of *DREF* altered by the temperature-sensitive mutation may be intrinsically required for maintaining the GSC state.

### DREF may act in concert with Mi-2/NuRD to regulate chromatin structure and gene expression

Genetic interaction studies uncovered a novel role for *Caf1* and *Mi-2*, components of the Nucleosome Remodeling and Deacetylase (NuRD) chromatin-modifying complex in repressing DREF-mediated self-renewal. Reducing the gene dosage of either *Caf1* or *Mi-2* function by half was able to rescue the GSC-loss phenotype in a *DREF*^*ts*^*/DREF*^*null*^ mutant background. Our results are consistent with previous studies indicating an antagonistic relationship between *DREF* and *Mi-2*. Reduction of *Mi-2* gene dosage by half had been shown previously to enhance defects caused by *DREF* overexpression in the eye, consistent with *Mi-2* antagonizing *DREF*[42]. Yeast two-hybrid screening identified the human homolog of Mi-2, CHD4, as a binding partner of human DREF and pull-down assays confirmed this association in Drosophila, showing that Mi-2 physically associates with DREF[42]. Mi-2 has been shown to interact with the DNA-binding domain of DREF, thereby inhibiting the ability of DREF to bind DNA in vitro[42]. Additionally, recent work with hDREF has shown that the reciprocal regulation as well: hDREF has been shown to increase SUMOylation of Mi-2 protein, thereby increasing dissociation of Mi-2 from chromatin[43].

Our data also indicated an inhibitory interaction between *DREF* and *CAF1*, raising the possibility that Mi-2 might act as a part of the NuRD complex to inhibit DREF function in male germline stem cells. The genetic interactions between *Mi-2* or *Caf1* and the *DREF*^*ts*^ allele suggest that Mi-2 and Caf1 can act as repressors of GSC self-renewal. Interestingly, we also found that *Mi-2* and *Caf1* are required for GSC maintenance in a genetic background wild-type for *DREF* function. The Mi-2/NuRD complex is known to play broad roles in reorganizing chromatin architecture to promote silencing[44], and Mi-2/NuRD has been shown to localize to hundreds of regions in the genome of Drosophila[42]. One possibility is that complete loss of Mi-2 function may cause general de-repression of genes important for cell-identity, self-renewal, and differentiation. Indeed, loss of Mi-2 in spermatocytes causes activation cryptic promoters at many sites in the genome, leading to massive misexpression[45]. It may be that Mi-2 plays a similar role in restraining misexpression during the early stages of germ cell development as well.

Previous studies suggest that Drosophila male GSCs are sensitive to changes in the transcript levels of genes required for self-renewal versus differentiation[41]. One role of DREF in Drosophila male GSCs may be to exclude Mi-2 from promoters of self-renewal genes, thereby allowing higher levels of expression at these loci. Under conditions of reduced DREF function in the DREF^ts^ mutant flies, Mi-2 may gain abnormal access to self-renewal genes, dialing down their expression and tilting the balance of GSC fate towards differentiation. We propose that in the context of *DREF*^*ts*^*/DREF*^*null*^ background, a half-dose reduction of *Mi-2* or *CAF1* function is sufficient to allow the partially functional DREF protein expressed in the *DREF*^*ts*^ mutant to overcome Mi-2-mediated repression of self-renewal genes and tilt the balance back towards GSC maintenance.

## Materials and methods

### Fly stocks and husbandry

All crosses were grown at 25°C on standard molasses media unless otherwise stated. DREF mutant alleles used in this study include 1*) al*^*1*^, *dp*^*ov1*^, *DREF*^*ts*^, *b*^*1*^, *pr*^*1*^, *FRT40A/SM6a* (derived from an EMS mutagenesis screen), 2) *DREF*^*KG09294*^ (from BDSC); this allele is a P-element insertion into the 5’UTR of *DREF* and has been previously reported to be a null allele that expresses little to no DREF protein[13], 3) *DREF*^*KG09294*^, *FRT40A* (from DGRC), 4) *Df(2L)BSC17* (referred to as *Df(DREF*), from BDSC) is a deletion that spans from *pelota* to *DREF*, and 5) *DREF*^*NP4719*^ (from DGRC), a P-element insertion in the 5’ UTR of DREF. Other mutant alleles used in this study include 1) *Caf1*^*9-2*^, a deletion allele of *Caf1* (gift from Joseph Lipsick, Stanford University[46]), 2*) Df(3R)BSC471* (referred to as *Df(Caf1)*, from BDSC), 3) *Mi-2*^*4*^ and *Mi-2*^*6*^ alleles (from BDSC and gift from J. Müller[47]), 4) *Df(3L)BSC445* (referred to as Df(Mi-2), from BDSC), 5) *Df(3R)BSC471* (referred to *as Df(Putzig)*, from BDSC), 6) *Df(3R) XNP*^*1*^ (referred to as *Df(XNP1)*, from BDSC), 7) *Df(3L)Exel7208* (referred to as *Df(Rpd3)*, from BDSC), 8) *Df(3R)Exel6153* (referred to as *Df(Mbdl)*, from BDSC) and 9) *Nurf301*^*4*^, a deletion allele of *Nurf301* (from BDSC). Other fly stocks used include 1*) al*^*1*^, *dp*^*ov1*^, *b*^*1*^, *pr*^*1*^, *FRT40A* (an isogenized version of FRT40A from BDSC), 2) *yw*, *hs-flp*^*122*^*; FRT40A*, *Ubi-GFP*, 3) *eyeless-Gal4*, *UAS-Flp; GMR-hid*, *2LCL FRT40A*(17), 3) *UAS-DEFL #6–1* [48] 4) *bam*::*bam-GFP*, a transgenic line driving *Bam-GFP* under the control of bam promoter (a gift from D. McKearin(21)) 5) *UAS-Upd* [49], and 6) *UAS-DREF*[22]. *al*, *dp*, *DREF*^*ts*^, *b*, *pr*, *FRT40A*, *UAS-DREF/SM6a* and *DREF*^*ts*^, *UAS-Upd* were generated by recombining *DREF*^*ts*^ chromosome onto the *UAS-DREF* or *UAS-Upd* chromosome, respectively. The following Gal4 drivers were used to drive UAS transgress in a cell-type specific manner, 1) *yw;;nanos-Gal4VP16* (a gift from R. Lehmann[50]), 2) *UAS-dicer2;;nanos-Gal4VP16*, 3*) yw;;Vasa-Gal4*, 4) *yw;;Bam-Gal4*, *UAS-dicer2*, (*Bam-Gal*4 lines was a gift from D. McKearin[21]) and 5) *C587-Gal4;tub-Gal80*^*ts*^;*UAS-dicer2* (C587-Gal4 line was a gift from S. Hou). RNAi lines for DREF (BDSC#35962), Caf-1 (BDSC#34069 and VDRC#105838) and Mi-2 (VDRC#107204 and 10766) were used in this study.

Temperature shift experiments for germ cell loss analysis in DREF transheterozygotes were performed by growing flies at 22°C until eclosion, and then shifting to 30°C on the day of eclosion. Dissections were performed on day of eclosion, or on one, two, or three days post eclosion.

For analysis of *DREF* function in the eye, clones were generated by crossing *DREF* alleles on FRT40A chromosomes to *eyeless-Gal4*, *UAS-Flp; GMR-hid*, *2LCL FRT40A/SM6a*. These crosses were grown at either 25°C, or at 30°C one day after the cross was set up.

### Immunofluorescence

Testes were dissected in 1X phosphate-buffered saline (PBS) and fixed in 4% paraformaldehyde diluted in PBS for 20 minutes at room temperature, washed twice in PBS with 0.1% TritonX-100, permeabilized in PBS with 0.3% TritonX-100 and 0.6% sodium deoxycholate for 30 minutes and blocked in PBS with 0.1% TritonX-100 and 3% bovine serum albumin for 30 minutes. Testes were incubated overnight at 4°C in primary antibodies against DREF (mouse, 1:100; gift from Dr. Andreas Hochheimer[28]), Vasa (goat, 1:50; Santa Cruz Biotechnology), Zfh-1 (rabbit, 1:5000; gift from R. Lehman), Traffic Jam (guinea pig, 1:5000; gift from D. Godt) LI ET AL NAT CELL BIOL 2003, Armadillo (mouse, 1:10; Developmental Studies Hybridoma Bank (DSHB)), DE-Cadherin (rat, 1:10; DSHB), FasIII (mouse, 1:10; DSHB), alpha-spectrin (mouse, 1:10; DSHB), Green Fluorescent protein (rabbit 1:400–1:1000; Invitrogen and Sheep 1:1000; Abd-Serotec), gamma-tubulin (mouse, 1:50; Sigma), phosphor-Histone3 Threonine3 (rabbit, 1:100; Upstate Biotechnology/Millipore), and STAT92E (rabbit, 1:100; gift from E. Bach[51]). Testes were incubated in appropriate secondary antibodies were from the Alexa Fluor-conjugated series (1:500; Molecular Probes) and mounted in VECTASHIELD medium containing DAPI to visualize DNA (Vector Labs). Tunel Assays were performed using the In Situ Cell Death Detection Kit, TMR red by Roche. Immunofluorescence images were taken using the Leica SP2 Confocal Laser scanning microscope. Phase and clonal analysis images were captured using a Zeiss Axioskop microscope and SPOT RT3 camera by Diagnostic Instruments or CoolSNAePz camera by Phomometrics. Images were processed using Adobe Photoshop and Illustrator CS6. Nuclear protein quantification was performed using ImageJ software and comparing the relative levels of the stained protein of interest (standardized to DAPI to control for sample depth) between experimental and control GSCs-.

### RNAi protocol

To perform RNAi knockdown in the germline, males from strains containing the RNAi hairpin of interest were crossed to UAS-dicer2;; nanos-Gal4VP16 virgins and grown at 25°C for 4 days after which the progeny of the cross were shifted to 30°C. Testes were isolated from males of the cross on the day of eclosion and 7 days post eclosion. In other cases, to follow the time course of stem cell loss, the progeny of crosses were grown at 18°C until eclosion, shifted to 30°C on the day of eclosion and males from the cross were dissected at different days post-shift to 30°C.

Somatic RNAi knockdown was performed by crossing the RNAi hairpin strains to virgin females containing the somatic lineage driver C587-Gal4;tub-Gal80^ts^;UAS-dicer2. Crosses were performed at 18°C until eclosion, when adults were shifted to 30°C and dissected 3- and 7-days post temperature shift.

Knockdown in transit amplifying cells and later stages was performed by crossing the RNAi hairpin to;; BamGal4, UAS-dicer2. Crosses were grown at 25°C for 4 days and then shifted to 29°C until eclosion and then they were dissected.

## Supporting information

S1 Fig *DREF* is required in the female germline. (A) Immunostaining of *DREF*^*ts*^*/Df(DREF)* mutant germaria (denoted by the bracket) which lacks vasa-positive germ cells. (B) DREF heterozygous germaria (bracket) contains vasa-positive germ cells. (C). Percentage of ovarioles containing empty germaria in *DREF*^*ts*^*/Df(DREF)* mutant germaria and sibling controls. Scalebar: 10 μm.
(TIF)

S2 Fig *DREF^ts^* vs *DREF^null^* protein Expression. (A-A’’’) *DREF*^*null*^ mutant GSCs (arrow) do not express DREF protein. (B-B’’’) *DREF*^*ts*^ mutant GSCs (arrow) still express DREF protein. Scalebar: 10 μm.
(TIF)

S3 Fig *DREF* RNAi using *bam-gal4* driver. (A) *DREF* RNAi driven under the BamGal4 driver in transit amplifying cells show dying cysts and an absence of meiotic and post-meiotic cell types (bracket). (B) Sibling hairpin only controls show meiotic and post-meiotic cell types (bracket). Scalebar: 50μm.
(TIF)

S4 Fig *CAF1* RNAi using *nanos-gal4vp16* driver. (A-A’’’) Caf1 RNAi driven in germline stem cells using NG4VP16 results in a loss of early germ cells after 5 days of RNAi expression at 30°C. (B-B’’’) Sibling hairpin only controls still retain GSCs under the same conditions. Scalebar: 20μm.
(TIF)
