# Supplementary figures and images for: DREF Genetically Counteracts Mi-2 and Caf1 to Regulate Adult Stem Cell Maintenance
Source: PLoS Genet. 2019 Jun 21;15(6):e1008187. doi: 10.1371/journal.pgen.1008187 (PMC6619835; doi:10.1371/journal.pgen.1008187)

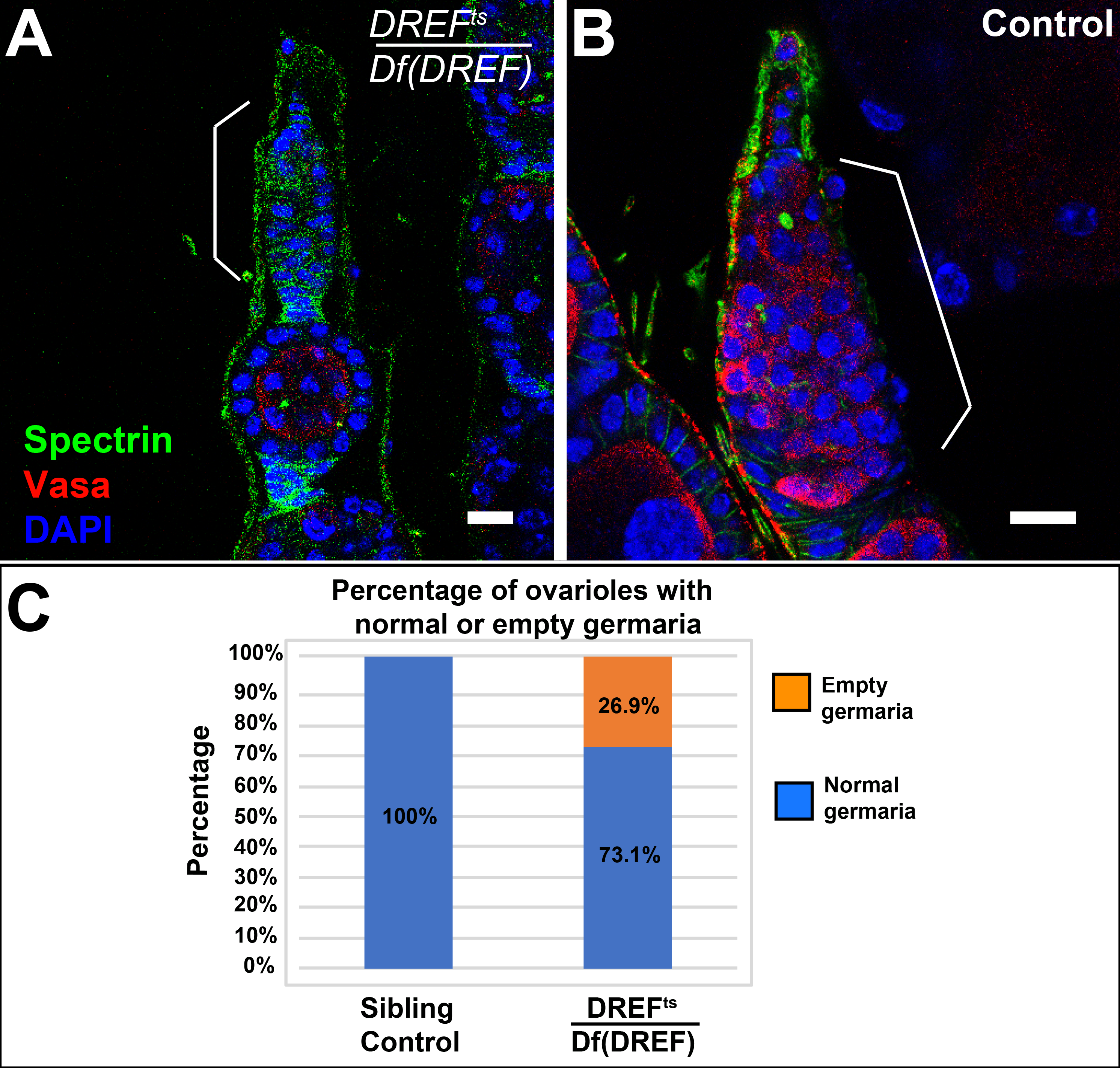

Supplement: S1 Fig — (A) Immunostaining of DREFts/Df(DREF) mutant germaria (denoted by the bracket) which lacks vasa-positive germ cells. (B) DREF heterozygous germaria (bracket) contains vasa-positive germ cells. (C). Percentage of ovarioles containing empty germaria in DREFts/Df(DREF) mutant germaria and sibling controls. Scalebar: 10 μm. (TIF) [file pgen.1008187.s001.tif]

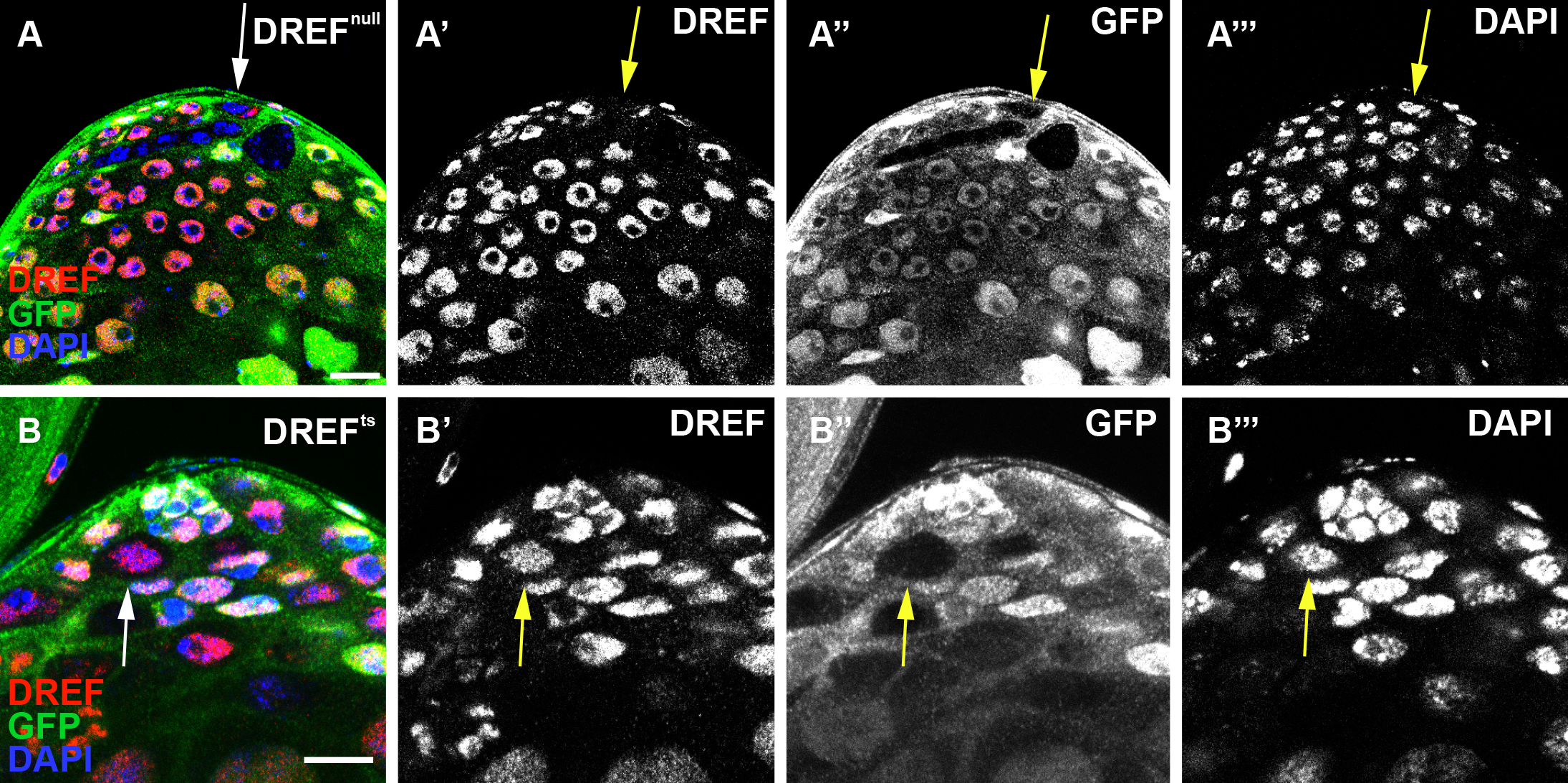

Supplement: S2 Fig — (A-A’’’) DREFnull mutant GSCs (arrow) do not express DREF protein. (B-B’’’) DREFts mutant GSCs (arrow) still express DREF protein. Scalebar: 10 μm. (TIF) [file pgen.1008187.s002.tif]

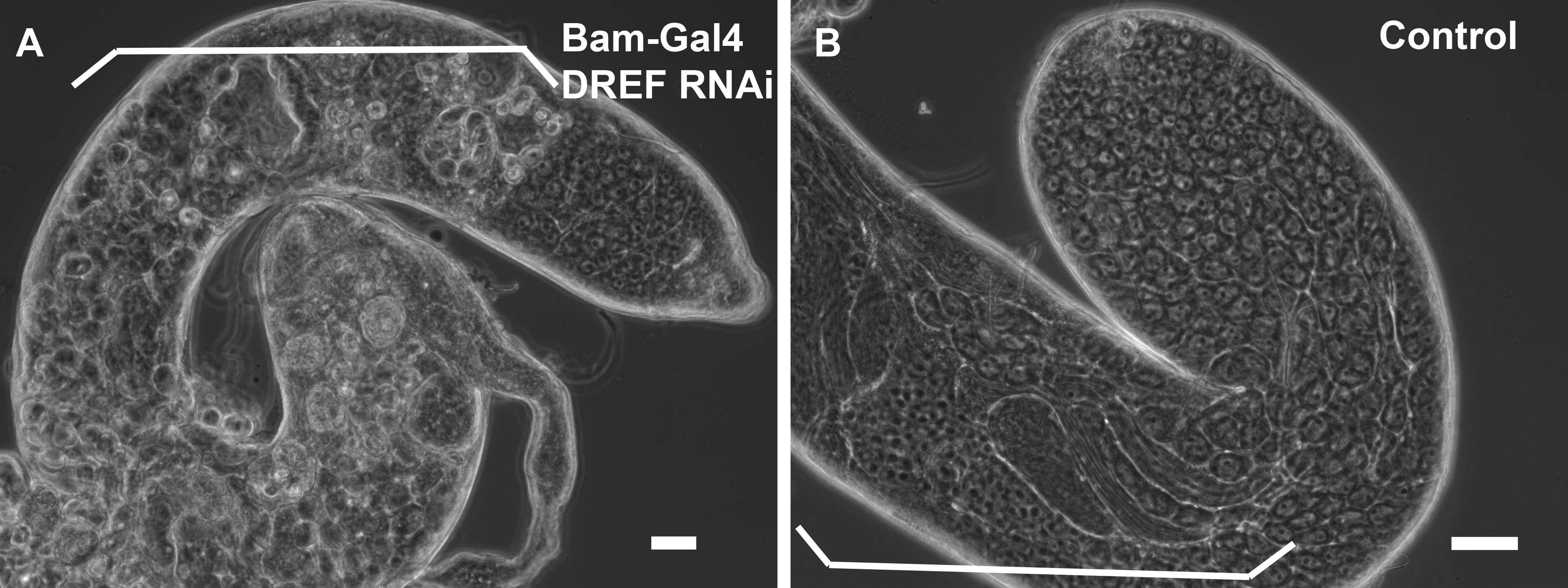

Supplement: S3 Fig — (A) DREF RNAi driven under the BamGal4 driver in transit amplifying cells show dying cysts and an absence of meiotic and post-meiotic cell types (bracket). (B) Sibling hairpin only controls show meiotic and post-meiotic cell types (bracket). Scalebar: 50μm. (TIF) [file pgen.1008187.s003.tif]

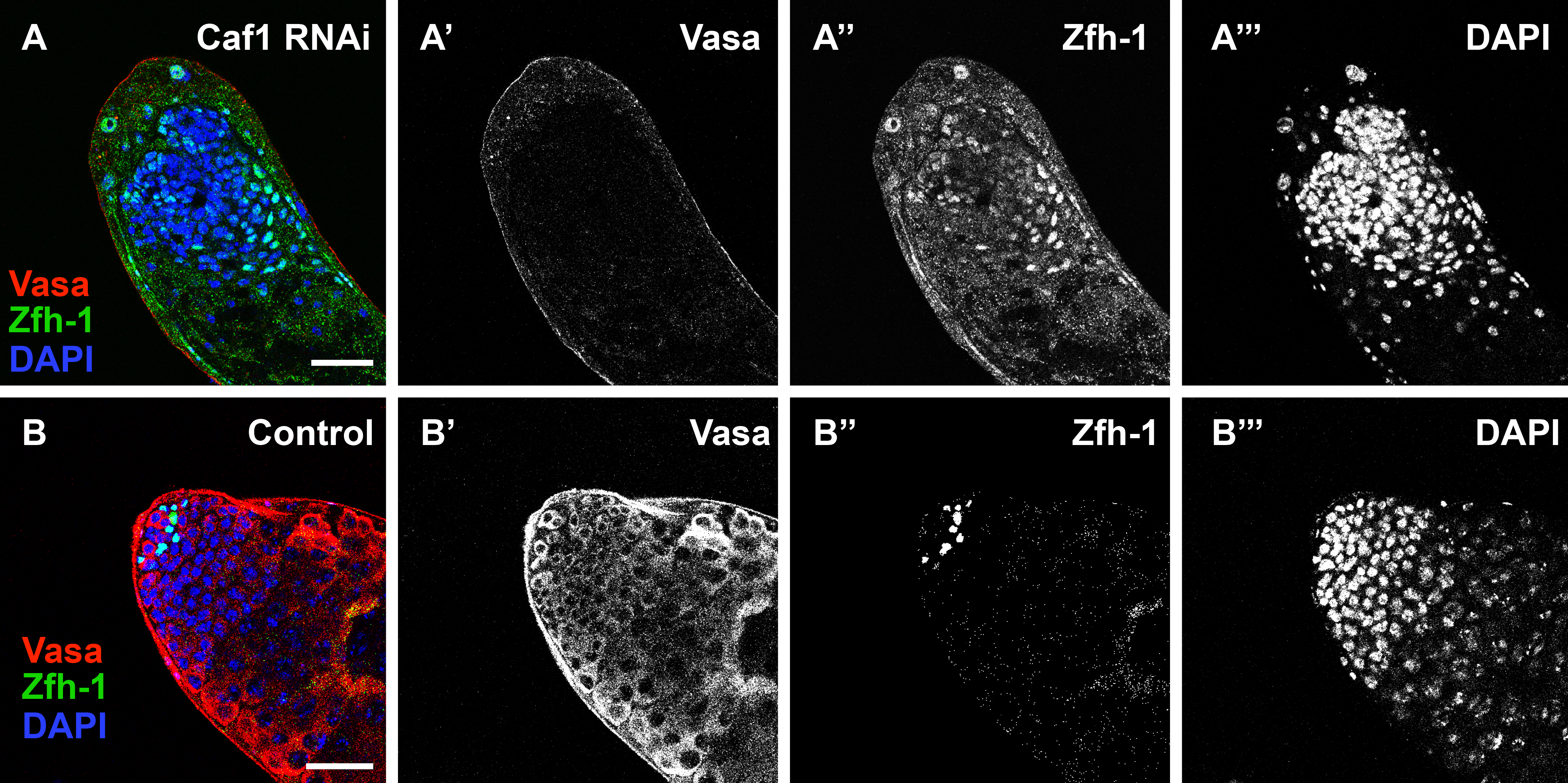

Supplement: S4 Fig — (A-A’’’) Caf1 RNAi driven in germline stem cells using NG4VP16 results in a loss of early germ cells after 5 days of RNAi expression at 30°C. (B-B’’’) Sibling hairpin only controls still retain GSCs under the same conditions. Scalebar: 20μm. (TIF) [file pgen.1008187.s004.tif]
